# Compressed Sensing/Sparse-Recovery Approach for Improved Range Resolution in Narrow-Band Radar

**DOI:** 10.1155/2016/3137146

**Published:** 2016-01-31

**Authors:** Sandra Costanzo

**Affiliations:** Dipartimento di Ingegneria Informatica, Modellistica, Elettronica e Sistemistica, Università della Calabria, 87036 Rende, Italy

## Abstract

A compressed sensing/sparse-recovery procedure is adopted to obtain enhanced range resolution capability from the processing of data acquired with narrow-band SFCW radars. A mathematical formulation for the proposed approach is reported and validity limitations are fully discussed, by demonstrating the ability to identify a great number of targets, up to 20, in the range direction. Both numerical and experimental validations are presented, by assuming also noise conditions. The proposed method can be usefully applied for the accurate detection of parameters with very small variations, such as those involved in the monitoring of soil deformations or biological objects.

## 1. Introduction

The application of radar devices for the remote detection and diagnostics of objects with high resolution capability is a strong focused point nowadays in many different contexts, going from soil deformation monitoring [1] to bioradiolocation [2], where surface displacements of just the order of millimeter need to be detected.

The most attractive configuration in these cases is given by the stepped-frequency continuous-wave (SFCW) radar [3], which transmits a series of narrow-band pulses at consecutive step Δ*f*, thus covering a wide overall bandwidth, even if adopting a narrow instant bandwidth. In this way, relatively high-range resolutions can be achieved by overcoming the disadvantages of wideband systems, such as high-speed Ads and fast processors [4]. As a further advantage, SFCW radar requires low transmit power, because the energy is spread out in time, thus avoiding nonlinear effects in the involved electronic components [5]. Nevertheless, two main drawbacks arise from the potential application of SFCW radar in the fast real-time detection of parameters with very small variations (e.g., biological objects); namely,

- low acquisition rate is typically provided, due to the slow scan over the radar bandwidth;
- conventional SFCW data processing, based on the application of the Inverse Fourier Transform (IFT) [6], gives a resolution which increases with the number of transmitted pulses, and this could be very high in those applications requiring precisions of the order of millimeter.

By exploiting or enforcing the sparsity nature of the scenario, a compressed sensing (CS [7])/sparse-recovery approach can be usefully adopted to enhance radar capabilities. Many representative works can be found in literature in this regards. A CS approach is adopted in [5, 8] to increase the data acquisition speed of an SFCW ground-penetrating radar, by exploiting the spatial sparsity of the target space to reduce the number of measurements required for an exact reconstruction. The sparse signal representation perspective is successfully considered in [9–11] to derive advanced image formation methods in the framework of synthetic aperture radar (SAR). In [12], CS is applied to SAR tomography reconstruction, by exploiting the sparsity of signals in order to achieve very high resolution in the elevation direction, but avoiding the adoption of huge elevation apertures. A sparsity factor between 1 and 4 (typically identifying the unknown positions in the elevation domain) is assumed in [12], and the new spectral estimation algorithm SL1MMER, based on CS, is introduced to mitigate the violation of the restricted isometry property (RIP) and the incoherence property [7], generally imposed to guarantee the accuracy of the sparsest solution. The effectiveness of the CS approach is also demonstrated in the framework of superresolution spectral estimation problem for three-dimensional SAR imaging [13], where the sparseness feature is exploited to reduce the number of multilook measurements (typically exhibiting reduced range and azimuth resolution) to achieve increased elevation resolution. All the cited works successfully discuss the superresolution power of the CS approach to identify a maximum number *N* = 3 targets in the elevation direction, but no resolution improvement is obtained in the range direction.

The possibility to enhance the radar resolution with CS has been recently highlighted in [14], where a sparseness target scene is assumed and simulation results reporting the estimation accuracy of the CS approach for a fixed discretization of the unambiguous range [6] into 100 grid points are discussed.

In this work, a detailed formulation of a CS-based processing algorithm able to identify close targets (in the range direction) by using narrow-band SFCW radar is presented. The effective range resolution enhancement is theoretically demonstrated, and a detailed discussion on the mathematical limits constraining the validity of the approach is presented, by revealing the possibility to identify a significant number of close targets (up to 20). Numerical simulations on both noiseless and Gaussian corrupted data are reported. Furthermore, experimental validations are presented on a real scenario composed by two metal plates (test targets) with a small separation distance of just 10 cm, accurately retrieved by adopting the CS-based processing algorithm to measured data obtained by a C-band (500 MHz) SFCW radar, fully designed at the Microwave Laboratory of University of Calabria.

## 2. Mathematical Formulation

With reference to a monostatic radar configuration [15], let us assume a transmitted signal with normalized amplitude, defined as follows:

(1) $$\begin{array}{c} t_{x}\left(\;t\;\right)=e^{j2\pi ft}. \end{array}$$

The signal received by the radar, as due to the interaction with a single target located in the field of view, can be expressed as follows:

(2) $$\begin{array}{c} r_{x}\left(\;t\;\right)=a\left(\;d\;\right)\cdot e^{j2\pi \left(t-\tau \right)}, \end{array}$$

where *d* is the target distance, *τ* = 2*d*/*c* is the echo delay, and *a*(*d*) is a factor depending on the target reflectivity and the free-space propagation losses [15].

The received signal (2) is mixed with a complex conjugate replica of the transmitted signal (1) to give [6]

(3) $$\begin{array}{c} s\left(\;f\;\right)=t_{x}\left(\;t\;\right)\cdot r_{x}^{*}\left(\;t\;\right)=a\left(\;d\;\right)\cdot e^{-j2\pi f\tau }. \end{array}$$

In the general case of *N* targets located in the radar field of view, the following expression can be derived for (3):

(4) $$\begin{array}{c} s\left(\;f\;\right)=\overset{N}{\underset{n=1}{\sum }}s_{n}\left(\;f\;\right)=\overset{N}{\underset{n=1}{\sum }}a_{n}\cdot e^{-j2\pi f\tau_{n}}, \end{array}$$

where *a*_*n*_ = *a*(*d*_*n*_), *n* = 1,…, *N*.

After applying the IFT to (4), the following result is obtained:

(5) StF−1sf=∫−∞+∞∑n=1Nan·e−j2πfτn·ej2πftdf=∑n=1Nan·δt−τn,

where *δ*(⋯) denotes the Dirac function.

Equation (5) provides the radar range profile in terms of elementary impulses sequence, whose locations identify the targets distances.

When adopting an SFCW radar, only *M* discrete samples of function *s*(*f*) are measured, at discrete frequencies *f*_*m*_, *m* = 1,…, *M*. As a matter of fact, a single sinusoid at fixed frequency is transmitted, received, and processed for each acquisition step. If assuming a set of frequencies with uniform spacing Δ*f*, the total bandwidth useful for the radar operation is equal to *B* = *M*Δ*f*, and the sampling period to be adopted, according to Shannon-Nyquist theorem, is given as follows:

(6) $$\begin{array}{c} \Delta t=\frac{1}{2B}=\frac{1}{2M\Delta f}=\frac{\Delta R}{c}, \end{array}$$

*c* being the free-space velocity.

From (6), the range resolution Δ*R* of SFCW radar can be derived as follows:

(7) $$\begin{array}{c} \Delta R=\frac{c}{2B}. \end{array}$$

This latter equation, well known in radar theory [11], shows that high resolutions (low values of Δ*R*) require high operating bandwidth *B*, thus strongly limiting the real applications of SFCW radar. As a matter of fact, in its actual data processing form, this kind of radar cannot be easily applied in those situations requiring range resolutions of the order of millimeter or below, such as in biomedical monitoring, where the radar should operate with a frequency bandwidth of the order of GHz.

Now, let us express (5) in the form of a discretized linear system of the kind:

(8) $$\begin{array}{c} {\underline{\tau }}_{M}=F^{-1}\left\{\;{\underline{s}}_{M}\left(\;f\;\right)\;\right\}. \end{array}$$

It is easy to observe that system (8) well fits a CS representation. As a matter of fact, data are collected in an orthogonal basis (Fourier series), and the left-hand side of (8) is a *N*-sparse vector, *N* being the number of targets.

When applying the Fourier Transform (FT) operator to both sides of (8), we obtain

(9) $$\begin{array}{c} {\underline{s}}_{M}\left(\;f\;\right)=F\left\{\;{\underline{\tau }}_{M}\;\right\}. \end{array}$$

If imposing a range resolution Δ*R*_*Q*_ < Δ*R*, the following undetermined system can be defined:

(10) $$\begin{array}{c} {\underline{s}}_{M}\left(\;f\;\right)=\underline{\underline{C}}\cdot F\left\{\;{\underline{\tau }}_{Q}\;\right\}, \end{array}$$

where

(11) C__=I__M×M ∣ O__Q−M×Q−M,Q=RuΔRQ,

*R*_*u*_ = *c*/2Δ*f* being the radar unambiguous range [11].

It must be noted that the number of data *M* remains fixed into (10); thus, the effective bandwidth for radar operation is again equal to *B* = *M*Δ*f*; however, a larger bandwidth is numerically defined by the introduction of additional (*Q* − *M*) elements into vector ${\underline{\tau }}_{Q}$ of (10). This leads to enhanced range resolution, as provided by (7) when imposing *B* = *Q*Δ*f*.

In the realistic case where noise is added to data, the sparsest solution of undetermined system (10) comes from constraining the *l*_2_-norm of the error to be less than some threshold *ε*:

(12) $$\begin{array}{c} \min{\left\|\;\underline{\tau }\;\right\|}_{L_{1}}:{\left\|\;\underline{s}-\underline{\underline{C}}\cdot F\left\{\;\underline{\tau }\;\right\}\;\right\|}_{L_{2}}<\varepsilon , \end{array}$$

where *ε* is a small positive number.

The first condition in (12) involves the minimization of the *l*_1_-norm of $\underline{\tau }$, thus enforcing its sparseness. The second condition ensures the solution to be consistent with data $\underline{s}$. Such recovery problem has been introduced in [16] under the name of basis pursuit for sparse coding.

It should be noted that (9) represents a unitary transformation, due to the presence of FT; thus, a stable transformation is obtained, which is the best working condition of CS formulation. However, some important mathematical aspects should be carefully analyzed to guarantee the validity of the CS approach expressed by (12). First of all, parameters *M*, *N*, and *Q* must be constrained to satisfy the following condition [12]:

(13) $$\begin{array}{c} M>O\left(\;N\log\frac{Q}{N}\;\right). \end{array}$$

As a matter of fact, under limit (13) the convex *l*_1_-norm minimization provides the same solution as the N-P hard *l*_*o*_-norm minimization.

When applying constraint (13) to our context case, a direct dependence from the SFCW radar parameters can be derived. As a matter of fact, let us replace into (13) the definitions of parameters *M*, *Q*; namely,

(14) M=BΔf,

(15) Q=RuΔRQ=c2ΔfΔRQ.

If imposing that

(16) $$\begin{array}{c} \Delta R_{Q}=\frac{\Delta R}{\alpha } \end{array}$$

*α* being an integer number (*α* > 1), the following simplified expression can be easily derived for parameter *Q*:

(17) $$\begin{array}{c} Q=\frac{c}{2\Delta f}\cdot \frac{2B}{c}\cdot \alpha =\frac{B}{\Delta f}\cdot \alpha =M\alpha . \end{array}$$

Then, if substituting (14) and (17) into (13), we obtain

(18) $$\begin{array}{c} \frac{B}{\Delta f}>O\left(\;N\log\left(\;\frac{B}{\Delta f}\cdot \frac{1}{N}\cdot \alpha \;\right)\;\right). \end{array}$$

This latter expression suggests that, once fixing the radar operating bandwidth *B* and the unambiguous range *R*_*u*_ (leading in turn to a prescribed spacing Δ*f*), a limit is imposed by (18) to the maximum number of targets which can be solved by the CS approach (12), with a range resolution Δ*R*_*Q*_ = Δ*R*/*α*.

**Figure 1:**
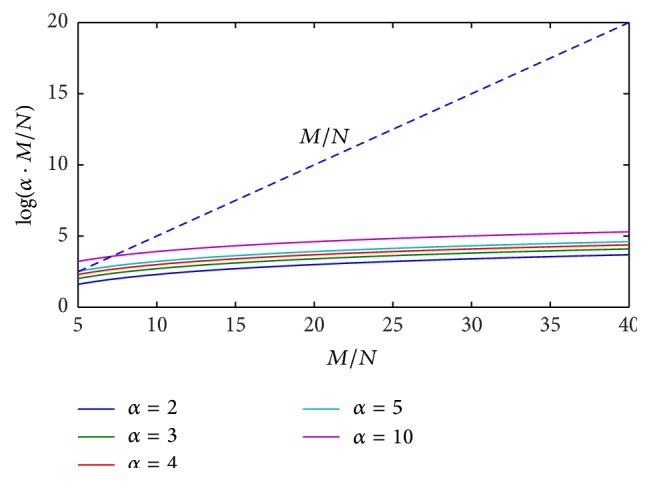
Behavior of (18) for the case *N* = 2, *α* = 2,3, 4,5, 10: ratio *M*/*N* is required to be between 7 and 10.

**Figure 2 fig2:**
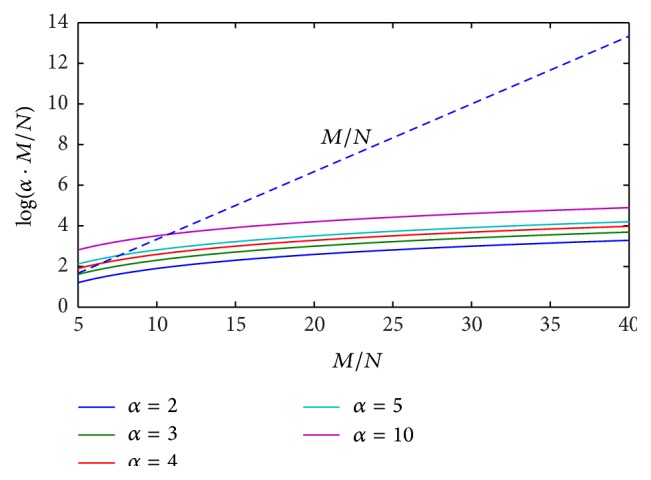
Behavior of (18) for the case *N* = 3, *α* = 2,3, 4,5, 10: ratio *M*/*N* is required to be between 11 and 15.

**Figure 3 fig3:**
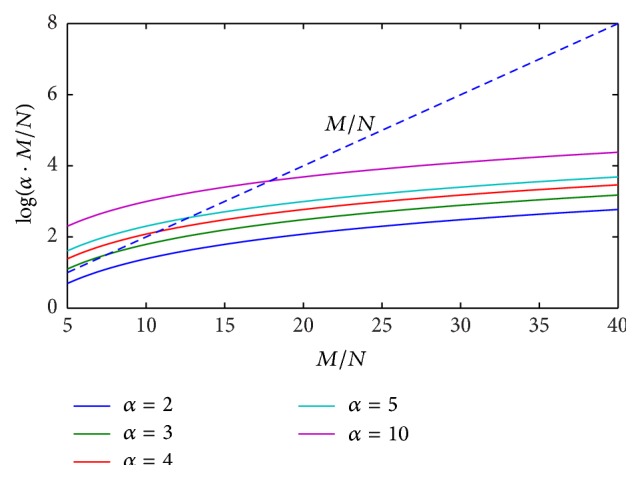
Behavior of (18) for the case *N* = 5, *α* = 2,3, 4,5, 10: ratio *M*/*N* is required to be between 18 and 20.

**Figure 4:**
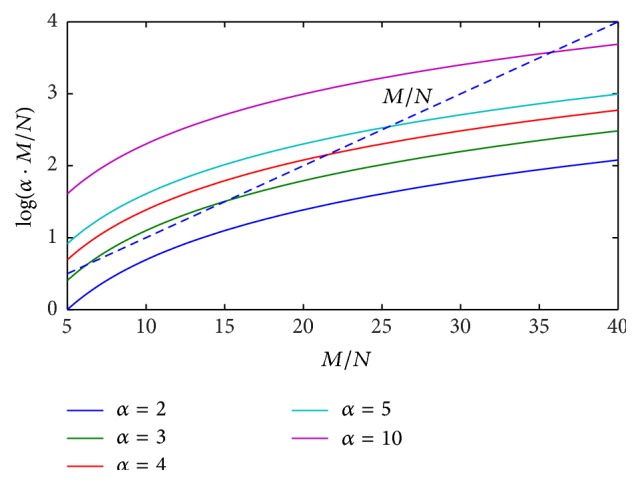
Behavior of (18) for the case *N* = 10, *α* = 2,3, 4,5, 10: ratio *M*/*N* is required to be between 36 and 40.

In order to highlight the above mathematical limitations of the proposed CS approach, numerical simulations of (18) are performed, by reporting the behavior of the right-hand term log⁡(*α* · (*M*/*N*)) versus the ratio *M*/*N*, for different values of parameter *α*. Results coming from numerical tests are illustrated in Figures 1 [Fig fig2] [Fig fig3]–4 for the cases *N* = 2, 3, 5, 10 and typical values of the ratio *M*/*N*.

It can be easily observed that, in the presence of a reduced number of targets to be solved (*N* = 2, 3), (18) is always satisfied by imposing a value of the ratio *M*/*N* between 7 and 10. When increasing the number of targets to be identified (*N* = 5, 10), greater values of the ratio *M*/*N* are required, depending on the imposed range resolution (value of parameter *α*). In the real cases, the hardware configuration of SFCW radar system limits the maximum ratio *M*/*N*, thus constraining the maximum attainable range resolution and the maximum number of resolvable targets, on the basis of (18).

Another important aspect, strongly influencing the validity of the CS approach (12), is that related to the choice of the regularization parameter *ε*, which establishes the tradeoff between the sparsity of the solution and the closeness of the solution to the measured data [13]. In the real case of noisy scenario, the value of parameter *ε* controlling the solution of (12) is quite critical and strictly related to the signal-to-noise (SNR) ratio, thus limiting in practice the “possible” maximum range separation to be detectable. When a Gaussian noise with zero mean and variance *σ* ^2^ is added to data, the expression suggested for the “optimal” regularization factor is given as follows [17]:

(19) $$\begin{array}{c} \varepsilon =\sqrt{2\log M}\sigma . \end{array}$$

The above choice is considered in the present work to perform the numerical tests discussed in the next section.

## 3. Numerical Simulations

In order to assess the validity of the CS approach outlined in Section 2, preliminary experiments are performed on simulated data. A radar bandwidth *B* = 500 MHz, providing a standard range resolution Δ*R* = 30 cm (7), and a spacing Δ*f* = 15 MHz, giving an unambiguous range *R*_*u*_ = 10 m, are assumed in the numerical tests.

**Figure 5:**
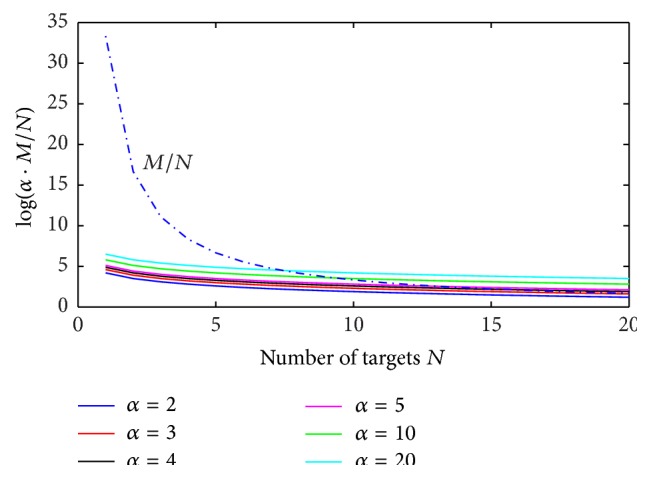
Behavior of (18) for the case *B* = 500 MHz, Δ*f* = 15 MHz.

As a first step, (18) is numerically simulated, as a function of the number *N* of targets, for different values of parameter *α*, thus obtaining the curves illustrated in Figure 5.

From the above simulations, it can be easily observed that, for values of parameter *α* between 2 and 4 (Δ*R*/4 ≤ Δ*R*_*Q*_ ≤ Δ*R*/2), the CS procedure guarantees the identification of a great number of targets, up to 20. When further increasing the resolution Δ*R*_*Q*_(10 ≤ *α* ≤ 20) a significant number of targets between 7 and 10 is again identified. This is a strongly enhanced achievement when compared to the existing results in literature, such as those reported in [12, 13], where the superresolution power of the CS approach is successfully applied to identify a maximum number *N* = 3 of targets in the elevation direction, but no resolution improvement is obtained in the range direction.

In order to demonstrate the range resolution enhancement offered by the proposed CS technique, two different target scenarios are assumed, including, respectively, a number *N* = 4 and 6 of targets. In both cases, a spacing between the targets less than the range resolution Δ*R* = 30 cm provided by the assumed radar configuration is considered. The range profile is computed by using the standard SFCW radar processing (IFT) as well as the CS procedure outlined in the present work. In this last case, the *l*_1_ minimization described by (12) is performed by assuming *α* = 3, thus imposing an improved range resolution Δ*R*_*Q*_ = 10 cm.

**Figure 6:**
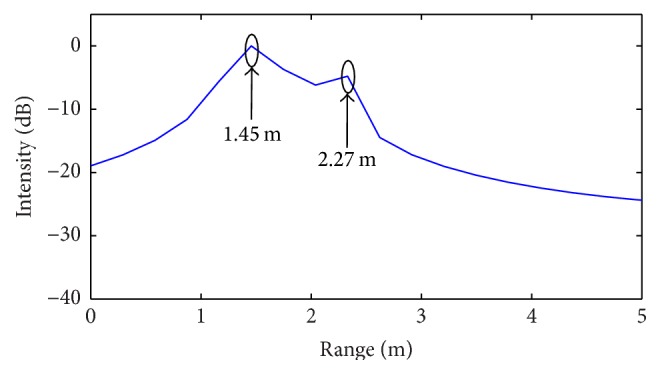
Range profile obtained from standard IFT algorithm (first scenario: *N* = 4 targets).

**Figure 7:**
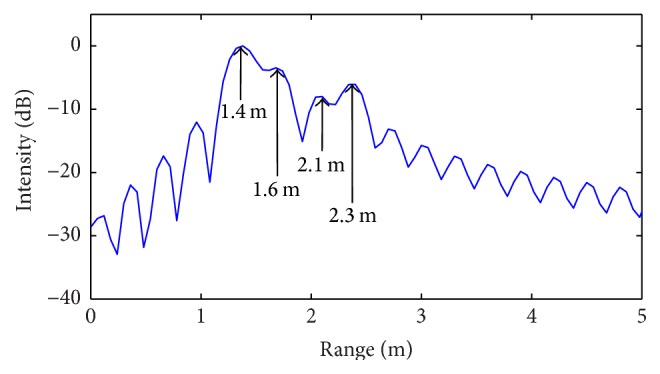
Range profile obtained from CS procedure (first scenario: *N* = 4 targets).

**Figure 8:**
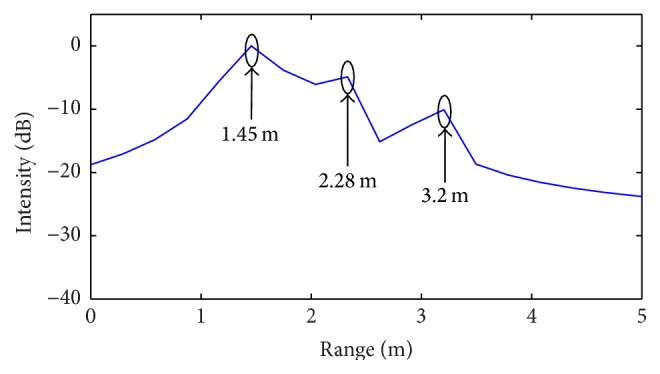
Range profile obtained from standard IFT algorithm (second scenario: *N* = 6 targets).

**Figure 9:**
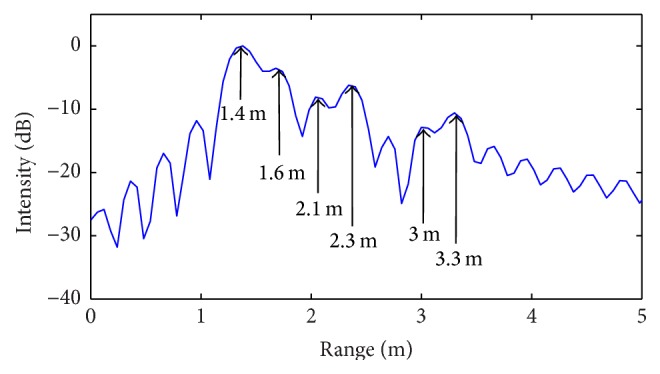
Range profile obtained from CS procedure (second scenario: *N* = 6 targets).

Firstly, ideal noiseless simulations are performed, whose corresponding results are illustrated in Figures 6–9.

By examining the above results, it can be easily observed that standard radar processing algorithm is able to identify only two targets (over four) for the first scenario (Figure 6) and only three targets (over six) for the second scenario (Figure 8). For both cases, the revealed positions are indicated by relative arrows. Meanwhile, the CS procedure properly identifies all targets in both simulated scenarios (Figures 7 and 9).

To verify the robustness of the proposed CS approach with respect to noisy data, additional simulations are performed by assuming the signals to be corrupted with additive Gaussian noise giving an SNR ratio equal to 20 dB. For these noisy tests, the *l*_1_ minimization is performed by imposing a regularization parameter *ε* satisfying (19).

**Figure 10:**
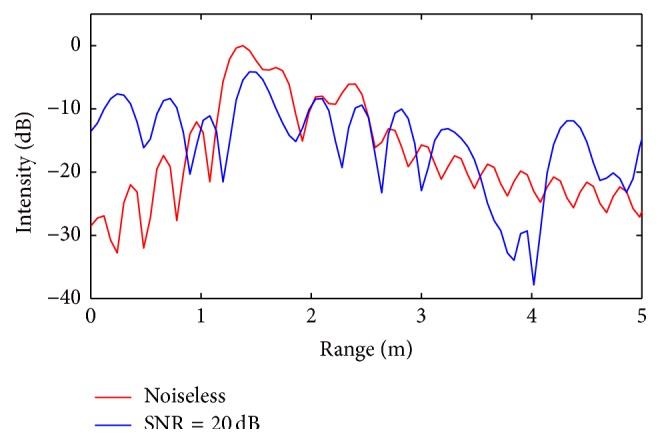
Range profile obtained from CS procedure (comparison between noiseless and Gaussian corrupted case, first scenario: 4 targets): only the second target is missed in the presence of noise (SNR = 20 dB).

**Figure 11:**
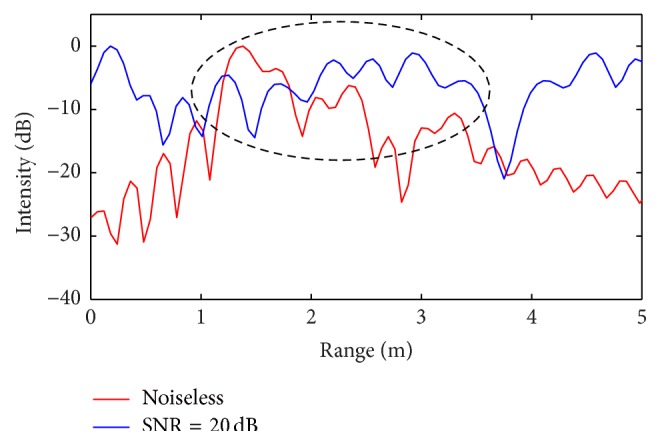
Range profile obtained from CS procedure (comparison between noiseless and Gaussian corrupted case, second scenario: 6 targets): all targets are revealed in the presence of noise (SNR = 20 dB).

The comparisons between the reconstructed radar range profiles with and without noise are reported in Figures 10 and 11, for the first and the second simulated scenario, respectively.

For the first scenario (Figure 10), the presence of noise leads to losing only one target, namely the second one, while in the second scenario all six targets positions are revealed, as indicated in the black dotted ellipse, even if the presence of additive noise increases the signal intensity.

## 4. Experimental Results

**Figure 12:**
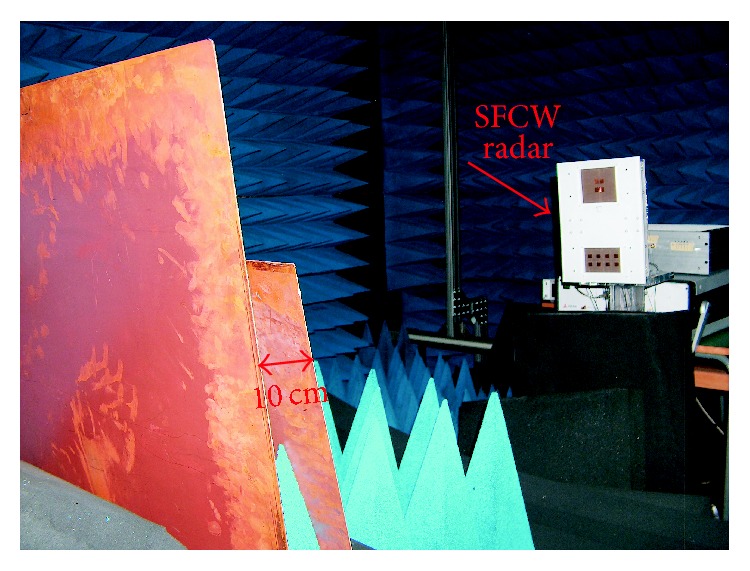
Test setup into the Microwave Laboratory at University of Calabria.

As a further assessment test, experimental validations on a laboratory noisy scenario are performed by adopting a C-band SFCW radar fully designed and realized at the Microwave Laboratory of the University of Calabria, in the framework of project PON 01-01503 for landslides monitoring, financed by the Italian Ministry of University and Research. The radar is characterized by an operating bandwidth *B* = 500 MHz, the same as that adopted for numerical simulations and thus able to distinguish targets with a range resolution Δ*R* = 30 cm. A spacing Δ*f* = 15 MHz is considered, which provides an unambiguous range *R*_*u*_= 10 m. To prove the validity of CS enhanced range algorithm, radar tests are performed into the anechoic chamber of the Microwave Laboratory at University of Calabria, by assuming the presence of two metal plates at distances *d*_1_ = 1.20 m, *d*_2_ = 1.30 m from the radar antenna aperture (Figure 12), with a separation smaller than Δ*R*.

Prior to performing the tests, a preliminary calibration procedure is applied, in the absence of targets, to properly identify the reflection peak due to the overall radar system delay. It results in being positioned at a distance approximately equal to 5 m.

**Figure 13:**
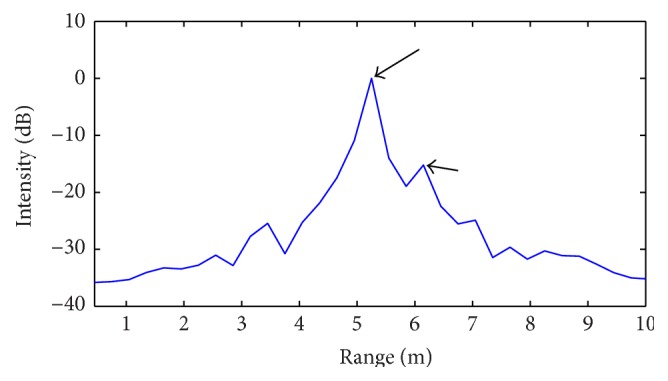
Radar intensity profile obtained from standard radar processing (IFT): experimental scenario of Figure 12.

When applying the standard (IFT) SFCW processing algorithm, the two targets are not distinguished but identified as a single object at a distance equal to 6.2 m (5 m + 1.2 m), as indicated by the arrows on the radar intensity profile of Figure 13.

The CS algorithm outlined in Section 2 is then applied by assuming *α* = 3 (Δ*R*_*Q*_ = Δ*R*/3) and thus able to guarantee, on the basis of (18), the identification of a maximum number *N* = 20 of targets separated by a minimum spacing Δ*R*_*Q*_ = 10 cm.

**Figure 14:**
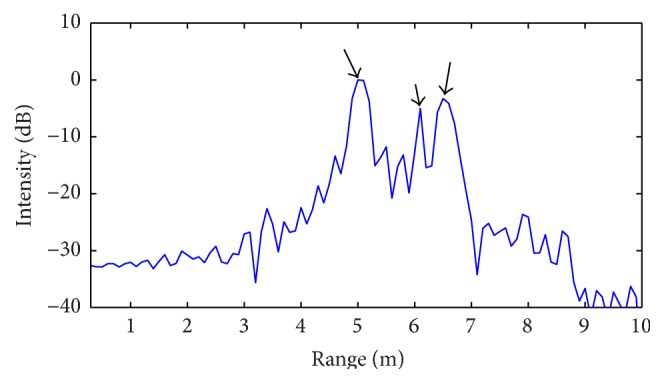
Radar intensity profile obtained from CS processing algorithm: experimental scenario of Figure 12.

As a matter of fact, the radar range profile in Figure 14, obtained as result of the CS procedure, properly contains two main peaks corresponding to the two targets positions. In both Figures 13 and 14, the peak associated with the overall delay of SFCW radar is also present at position equal to 5 m.

## 5. Conclusion and Future Work

The enhancement of range resolution, when adopting a CS approach for the processing of data coming from narrow-band radars, has been theoretically demonstrated, and a CS-based target reconstruction procedure has been properly formulated.

Mathematical aspects concerning the validity of the proposed sparse-recovery method are discussed, and the ability to reconstruct a great number of targets, up to 20, is demonstrated. Both numerical and experimental validations, illustrating the enhanced range profile reconstruction, have been reported. Concerning future work, the application of the proposed technique to fast and real-time monitoring of very small displacements, such as those occurring in soil deformations or bioradiolocation field, will be considered.
